# Comparative Analysis of Manual ELISA and Ella, an Automated Instrument for ELISA, in Measuring Serum Galectin-3 Levels in Breast Cancer Patient Samples

**DOI:** 10.3390/cancers17193206

**Published:** 2025-10-01

**Authors:** Ella G. Markalunas, Shannon E. Harold, David H. Arnold, Julie C. Martin, W. Jeffery Edenfield, Anna V. Blenda

**Affiliations:** 1 Department of Public Health, Brown University, Providence, RI 02912, USA; ella_markalunas@brown.edu; 2 Department of Biomedical Sciences, University of South Carolina School of Medicine Greenville, Greenville, SC 29605, USA; sharold@email.sc.edu (S.E.H.); dharnold@email.sc.edu (D.H.A.); 3 Prisma Health Cancer Institute, Prisma Health, Greenville, SC 29605, USA; julie.martin@prismahealth.org (J.C.M.); jeffery.edenfield@prismahealth.org (W.J.E.); 4 Department of Medicine, University of South Carolina School of Medicine Greenville, Greenville, SC 29605, USA

**Keywords:** ELISA, automated assay, Ella, galectin-3, breast cancer, Simple Plex assay, comparative analysis

## Abstract

**Simple Summary:**

Galectin-3 is a protein involved in the immune system that may be useful for future breast cancer treatments. Serum galectin-3 levels are usually measured using manual Enzyme-Linked Immunosorbent Assay (ELISA), but new automated tools, such as the Ella instrument, can perform ELISA more quickly and reliably. The goal of this paper is to see whether there is a difference in the measurements of serum galectin-3 between the manual ELISA and the new Ella instrument. Serum galectin-3 levels were measured in 95 breast cancer patients using both manual ELISA and automated Ella. Our study found that there is moderately good agreement between ELISA and Ella measurements, but Ella measurements tend to be lower than those of manual ELISA. The Ella instrument was also more precise, resulting in less variation in measurements of the same sample. This study helps clarify the differences between ELISA and Ella, which should be taken into consideration for measuring galectin-3 levels in breast cancer patient samples.

**Abstract:**

**Background**: Circulating galectin-3 (Gal-3) levels have been indicated as a promising diagnostic, prognostic, and therapeutic target in breast cancer patients. Specifically, serum galectin-3 levels are traditionally measured using manual Enzyme-Linked Immunosorbent Assay (ELISA), but recent automated methods, such as Simple Plex assay by ProteinSimple™ run on an Ella instrument, have shown promising evidence of being more efficient and less error-prone than manual methods. This paper aims to assess whether there are differences in serum galectin-3 measurements between manual and automated ELISA methods. **Methods**: Serum galectin-3 levels were initially analyzed from one hundred and fifteen breast cancer samples using both manual ELISA and the Ella instrument. Following coefficient of variation (CV) and outlier analysis, ninety-five samples were analyzed further with JMP statistical software to perform Shapiro-Wilk, Spearman’s correlation, Wilcoxon signed-rank, and regression analyses. **Results**: The Ella instrument resulted in significantly lower CV values, confirming that it is more precise and reliable than manual ELISA methods. There was a moderate correlation between ELISA and Ella measurements (r = 0.49, *p* < 0.0001), but a Wilcoxon signed-rank test revealed that serum gaelectin-3 measurements obtained with the Ella instrument were significantly lower compared to those obtained with manual ELISA, with a mean difference of −5.19 ng/mL (*p* < 0.0001). Regression analysis showed a significant increase in the difference between manual ELISA and Ella measurements as serum galectin-3 levels increase (*p* < 0.0001). This difference in measurements between manual and automated ELISA techniques remained consistent when analyses were performed within each breast cancer stage, immunophenotype, and histology. **Conclusions**: While the Ella instrument is a fast and reliable tool, the discrepancies between manual ELISA and the Ella instrument in quantifying serum galectin-3 levels are important to consider prior to widespread use.

## 1. Introduction

Galectins are a family of β-galactoside-binding proteins that modulate immune responses and tissue growth, differentiation, and regeneration [1]. There are three different types of galectin structure: prototypical, chimeric, and tandem-repeat. Of the 15 known mammalian galectins, galectin-3 is the only chimera-type galectin with a unique N-terminal extension and a single C-terminal carbohydrate recognition domain [2]. Figure 1 from Kamili et al. (2016) demonstrates the individual domains and the quaternary structure of the three different types of galectin structures [3].

Expression of galectin-3 has been identified in nearly every tissue, including lungs, colon, kidneys, blood, heart, urogenital tracts, reproductive tract, and digestive tracts [4,5]. Additionally, galectin-3 expression has been demonstrated in myeloid, epithelial, and endothelial cells [6]. Galectin-3 is localized within the cytoplasm, nucleus, cell membrane, and even extracellularly. However, some cell types exclusively or predominantly express the protein in either the nucleus or the cytoplasm [7]. Galectin-3 has been implicated across pathologies as an important biomarker and factor in disease processes. Utilizing galectin-3 as a prognostic factor and potential therapeutic target has shown promising results in many diseases, including diabetes, intracerebral hemorrhage, cardiovascular disease, kidney disease, and many types of cancers [8,9,10,11,12,13,14].

Galectins are highly involved in immune system regulation, and galectin-3 is a significant modulator of the signaling pathways governing immune system activation. Galectin-3 is an important modulator of T-cell growth, and previous studies suppressing galectin-3 levels have shown a significant reduction in T-cell proliferation [15,16]. Additionally, galectin-3 is involved in the activation of mast cells, monocytes, and macrophages as well as facilitating neutrophil invasion through a pro-inflammatory response [2,17,18,19]. Intracellularly, galectin-3 also acts as an important pre-mRNA splicing factor with galectin-1 [20]. Figure 2 from Dumic et al. (2006) details a few of the various roles that galectins play in immune system regulation [21].

In cancer cells, galectin-3 promotes cell survival, growth, and proliferation. Previous studies have consistently shown that galectin-3 is upregulated in many types of cancer, including breast cancer [13]. Galectin-3 is highly expressed in both natural and chemically induced tumors [22]. The exact mechanistic role of galectin-3 has not yet been determined, as galectin-3 is implicated in many signaling pathways involved in breast cancer disease progression, such as the Ras/Raf/MEK/ERK (Rat Sarcoma/Rapidly Accelerated Fibrosarcoma/Mitogen-Activated Protein Kinase Kinase/Extracellular Signal-Regulated Kinase) and Notch pathways, which modulate cell survival and metastasis [23]. Angiogenesis, a critical element in metastasis and tumor growth, is also heavily modulated by galectin-3 [24]. In endothelial cells, galectin-3 acts as a scaffolding protein, stabilizing and promoting the vascularization process [23]. Additionally, galectin-3’s activation of the Vascular Endothelial Growth Factor A Receptor 2 (VEGFR2) signal transduction pathway and Jagged-1/NOTCH-1 (Neurogenic Locus Notch Homolog Protein 1) activation is another mechanism for promoting angiogenesis and metastasis in breast cancers [25].

Galectin-3 plays another crucial role in chemoresistance and tumor survival. Under stressed conditions such as hypoxia and nutrient deficiency, where cancerous cell growth might normally be slowed, upregulation of galectin-3 promotes cell survival and migration to areas with more nutrients and oxygen [23]. When exposed to apoptotic agents such as nitric oxide, galectin-3 overexpression results in higher rates of survival for breast carcinoma cells. Mechanistically, galectin-3 promotes apoptosis resistance in this case by maintaining mitochondrial integrity through inhibition of cytochrome c release and caspase activation, as well as preventing the loss of cell adhesion [26]. Cisplatin, an important therapeutic drug that induces apoptosis in breast cancer cells, is inhibited by high levels of galectin-3 expression [27,28].

Within the tumor microenvironment, galectin-3 promotes immunosuppression by suppressing the proliferation of tumor-reactive T cells [29]. Intracellularly, galectin-3 inhibits chemotherapeutic-induced apoptosis in tumor cells, while extracellularly, galectin-3 promotes T-cell and thymocyte death by binding to surface glycoproteins [30,31]. Because of its role in immunosuppression, galectin-3 inhibitors have shown promise in early-phase clinical trials as potential immunotherapies against many cancer types [30].

Galectin-3 has also shown promise as a potential biomarker for disease progression within breast cancer patients. Galectin-3 cleavage is an active process during breast cancer progression, making it a potential indicator for tumor activity [32]. Elevated serum galectin-3 levels are associated with tumor progression and metastasis among all immunophenotypes of breast cancer [33]. However, galectin-3 levels vary across immunophenotypes, with noted upregulation in Triple Negative Breast Cancer [34]. Triple Negative Breast Cancer cells secrete galectin-3, resulting in immunosuppression by inhibiting CD45 (Leukocyte Common Antigen) signaling, which decreases oxidative phosphorylation, resulting in mitochondrial dysfunction in T cells [35].

Across breast cancer subtypes, certain cancer-critical gene mutations are correlated with an upregulation of galectin-3 levels, indicating the possibility of utilizing gene panels and galectin levels in tandem to guide treatment options [36,37]. Galectin-3 levels have also been found to be significantly elevated among patients with invasive ductal carcinoma compared to those with ductal carcinoma in situ [37]. Furthermore, there is greater expression of galectin-3 in micropapillary carcinomas compared with invasive ductal carcinomas [38].

With regard to stages, galectin-3 is elevated across all stages of breast cancer without significant differences between stages, indicating that galectin-3 dysregulation likely occurs early in disease progression [13]. Because of its role in metastasis and chemoresistance, galectin-3 is an important prognostic factor, and its accurate measurement in patients could serve as a guide for developing treatment plans and predicting treatment response.

In addition to its prognostic potential, galectin-3’s dysregulation and impact on innate immune response in breast cancer cells make it an important consideration for breast cancer treatment protocols. Consistent with other cancer types, galectin-3 promotes apoptotic resistance in tumor cells, limiting the effectiveness of apoptotic therapies. However, galectin-3 knockdown resensitizes breast cancer cells to these treatment mechanisms [34]. In B cells, galectin-3 promotes Interleukin-10 (IL-10) production, which impairs antitumor immunity. One study found that combining galectin-3 inhibition with chemotherapy reduces tumor growth and improves antitumor immunity in breast cancer patients [39]. Preliminary studies have also found promising evidence for the potential use of galectin-3 as an isolated therapeutic target. For example, treating mice with breast cancer tumors with a truncated form of galectin-3 reduced the frequency of metastases and overall tumor volumes and weights [40]. Due to the myriad of potential uses for galectin-3 in breast cancer treatment, it is imperative to understand how utilizing different methodologies for measuring serum galectin-3 levels has the potential to impact the results.

ELISA is the traditional method for measuring galectin-3 levels. The four major types of ELISA are direct, indirect, sandwich, and competitive ELISA [41]. There are commercial galectin kits available that utilize each of the types of ELISA, but sandwich ELISA, which “sandwiches” the target antigen between capture and detection layers of antibodies, is the most frequently employed [42]. Each type of ELISA presents different disadvantages, such as low sensitivity, high cost, time, cross-reactivity with secondary detection antibodies, and large sample requirements [43]. Manual ELISA introduces variability in results due to human error, including pipetting errors, contamination, incubation time, or temperature differences [44]. The complexity and number of steps involved in manual ELISA result in decreased reliability between tests and testers. Due to limitations in the precision of results as well as the high time and sample requirements, automated instruments have been developed to carry out ELISA tests.

Researchers are increasingly moving towards automated assays for measuring galectin-3 in favor of efficiency and reliability. The ARCHITECT assay has proven to be both cost-effective and reliable in producing results similar to those in traditional manual ELISA when measuring serum galectin-3 levels in heart failure patients [45,46]. ProteinSimple™, under Bio-Techne^®^, has developed an automated immunoassay platform called Simple Plex, which is run on an Ella instrument. Ella is capable of running a simultaneous analysis of up to eight analytes and up to seventy-two samples in a single run [47]. Due to its automated nature, Ella reduces sample volume, run time, and manual steps, which not only increases efficiency but also reduces potential sources of error [48]. Each cartridge features a built-in calibration curve, eliminating the need for manual creation of calibration curves, which is typically required for traditional ELISA. The Simple Plex runner then automatically calculates the concentrations of the desired target from these calibration curves. In traditional ELISA, multiplexing, measuring multiple analytes in a single experiment, usually reduces the sensitivity of the measurements due to cross-reactivity between analytes [47]. The microfluidic channels of the Ella cartridges separate each analyte during multiplexing, which eliminates the possibility of cross-reactivity and maintains a high level of sensitivity [49]. Previous studies have shown that Simple Plex analysis with Ella maintains a high level of reproducibility between days and users, making it a highly reliable analysis method [47].

While comparing serum galectin-3 levels measured with manual ELISA compared with the automated Simple Plex analysis on Ella has yet to be done, Ella has shown promising results when measuring other biomarkers. In healthy volunteers, ELISA and Ella showed good concordance in measuring Monocyte Chemoattractant Protein-1 (MCP-1)/Chemokine (C-C motif) Ligand 2 (CCL2), Vascular Endothelial Growth Factor A (VEGF-A), Tumor Necrosis Factor Alpha (TNF-α), and Interleukin-6 (IL-6) levels [48]. Additionally, Ella possesses higher sensitivity than manual ELISA while minimizing the sample, time, and manual step requirements, as well as test-retest and user variability. Another study found agreement between manual ELISA and Ella in immunoglobulin G and antidrug antibody quantification [49]. This study noted that Ella possesses a wider assay range, facilitating a more dynamic range of measurements that it can collect. Additionally, ELISA and Ella possess similar levels of precision in the drug quantification assay, but Ella exhibits significantly higher precision in the immune complex assay. Within healthy women participating in breast cancer prevention trials, adiponectin and leptin levels showed good agreement between manual ELISA and automated Ella measurements [50]. However, as the leptin measurements increased, there was a greater difference between ELISA and Ella measurements, with Ella measurements being greater on average than ELISA measurements. Similarly, as adiponectin measurements increased, there was a greater difference between ELISA and Ella measurements, with Ella measurements being lower on average than ELISA measurements at high adiponectin levels. This indicates a possible overestimation of leptin levels and an underestimation of adiponectin levels by the Ella instrument at higher concentrations [50]. These variations in ELISA and Ella measurements highlight the importance of understanding the comparisons between performing ELISA and Ella to ensure accurate data collection for patients.

This paper aims to compare the serum galectin-3 levels of breast cancer patients using manual ELISA and automated Simple Plex with the Ella instrument. This addresses a gap in current research related to the comparison between manual ELISA and automated ELISA using an Ella instrument when measuring galectin-3 levels in breast cancer patients. With clinical researchers increasingly looking towards galectins as potential cancer biomarkers or therapy targets, it is imperative to understand the differences between these two methods.

## 2. Materials and Methods

### 2.1. Patient Sample Collection

One hundred and fifteen breast cancer serum samples were obtained from Prisma Health Cancer Institute’s (PHCI) Biorepository. Patients signed a consent form at the time of sample collection, and sample collection was performed at the same time as necessary clinical procedures. The collection years ranged from 2013 to 2022. The PHCI manages specimen collection, handling, storage, and processing, as well as equipment maintenance and processing. The PHCI biorepository houses serum samples stored at −80 °C in multiple aliquots to avoid multiple freeze-thaw cycles. The biorepository data, including patient sample data, are included in the Table S1.

### 2.2. Measurement of Serum Galectin-3 Levels Using ELISA Technology

Serum galectin-3 levels were measured in July of 2024 for all one hundred and fifteen samples using enzyme-linked immunosorbent assay (ELISA) kits from R&D Systems (Minneapolis, MN, USA). This is a solid-phase sandwich ELISA kit performed in duplicate for all samples. This kit utilizes raised antibodies to detect the concentration of serum galectin-3 levels. Protein concentrations were evaluated using a microplate reader and a manually created standard curve. Ninety-six samples had coefficients of variation (CV) less than or equal to 10%. There was one outlier that was removed to avoid skewed data because this datapoint was over 40 ng/mL from the next closest serum galectin-3 concentration, so ninety-five samples were included for the data analysis.

### 2.3. Measurement of Serum Galectin-3 Levels Using the Automated Ella System

Serum galectin-3 levels were also analyzed for all one hundred and fifteen samples at the same time as ELISA measurements in July 2024 using Simple Plex human galectin-3 cartridges on an Ella instrument, both from ProteinSimple™ (San Jose, CA, USA) within Bio-Techne^®^ (Minneapolis, MN, USA). The Ella system uses microfluidic assay cartridges capable of assessing up to eight analytes simultaneously. By separating each target into separate channels, the system prevents cross-reactivity. The Ella instrument uses a factory-generated, pre-loaded standard curve to automatically assess the protein concentrations using fluorescence. Assessment was performed in triplicate for all samples. All samples had a CV of less than 10%.

### 2.4. Data Analysis

Data analysis was performed using JMP Pro 17, a software developed by the SAS Institute (Cary, NC, USA). Figures were generated using Prism, which is a part of GraphPad Software Version 10.4.2 (Boston, MA, USA). In the summary plots, means, standard deviations (SD), medians, interquartile ranges (IQR), and ranges are reported to provide an overview of the data distribution. Normality of both ELISA and Ella measurements of galectin was assessed using the Shapiro-Wilk test. Samples that satisfied normal conditions were analyzed using Pearson’s correlation and a paired *t*-test. Samples that were not normally distributed were analyzed using Spearman’s correlation and a Wilcoxon signed-rank test. A Wilcoxon signed-rank test pairs each ELISA measurement with the corresponding Ella measurement from the same sample. In samples where normality conditions were satisfied but the sample contained twenty or fewer samples, nonparametric statistical analyses were performed to ensure a stringent analysis. Samples were also compared using a least-squares regression line, and a Bland-Altman plot was used to compare the differences between the Ella instrument and the established standard for serum galectin-3 level measurements, manual ELISA.

## 3. Results

### 3.1. Characterization of Breast Cancer Patient Sample Group

Table 1 characterizes the clinical distribution of all one hundred and fifteen samples across stages, histologies, and immunophenotypes. Immunophenotypes were determined based on the presence of hormone receptors, the Human Epidermal Growth Factor Receptor 2 (HER2) protein levels, and Ki67 scores, which measure the percentage of cancer cells that are actively dividing. Luminal A is defined as estrogen and/or progesterone receptor positive but HER2 negative with low Ki67 scores. Luminal B is estrogen receptor positive and HER2 negative with high Ki67 scores. Luminal B-like has consistently high Ki67 scores, but it is also HER2 positive. HER2 enriched is hormone receptor negative and HER2 positive. Finally, Triple Negative is hormone receptor and HER2 negative [51]. Immunophenotype data were only available for seventy-nine of the samples. For subtype analysis, the sample of ninety-five subjects was utilized as discussed in Section 3.3.

### 3.2. Measurement Characteristics of Galectin-3 in ELISA and Ella Platforms

A previous study recruited a healthy population of 533 people, and after testing for preexisting conditions that might confound results, they developed a sample of 180 healthy subjects. Within this sample, researchers found that 95% of galectin-3 levels fall between 5.9 and 18.1 ng/mL [52]. Another study of over a thousand subjects identified an upper limit for healthy galectin-3 levels at 22.1 ng/mL [53]. Table 2 shows the overall distribution of serum galectin-3 levels determined using the two techniques for the ninety-five samples with a CV less than 10% from our study. Due to the lack of normal distribution, the median and interquartile range (IQR) are the most representative statistics for summarizing the ELISA and Ella measurements of serum galectin-3 levels in our sample. The median ELISA measurement of serum galectin-3 levels (N = 95) was 10.59 ng/mL with an IQR of 7.43–15.10 ng/mL. The median Ella measurement of serum galectin-3 levels (N = 95) was 6.70 ng/mL with an IQR of 5.20–8.80 ng/mL. The median difference between ELISA and Ella measurements of serum galectin-3 levels for the same subjects was −4.32 ng/mL with an IQR of −8.54 to −0.30.

### 3.3. Quantification of Assay Precision

When performing ELISA analysis, coefficient of variation (CV) values are used to quantify the variability in measurements due to the duplicate or triplicate nature of such analyses. Lower CV values indicate higher reproducibility and higher precision. A coefficient of variation less than 10% is the typical standard for intra-assay values, and a CV less than 15% is typical for inter-assay values. Majority (83.4%) of ELISA measurements (N = 96) had a CV value less than 10% while all Ella measurements had CV values less than 10%. The normal distribution of CV values for ELISA and Ella was tested using the Shapiro-Wilk Test. Both ELISA’s CV values and ELLA’s CV values were found not to be normally distributed with *p*-values < 0.0001. A Wilcoxon Signed Rank Test was performed to compare ELISA and Ella CV values for each patient. On average, Ella resulted in significantly lower CV values than manual ELISA with a mean difference of 5.08% (*p* < 0.001). Table 3 shows the distribution of CV values between measurements with manual ELISA and automated Ella techniques. Due to the lack of normal distribution, the median and interquartile range (IQR) are the most representative statistics for summarizing the CV values of the ELISA and Ella measurements. The median ELISA CV value (N = 115) was 4.75% with an IQR of 1.85–7.82%. The median Ella CV value (N = 115) was 2.10% with an IQR of 1.37–3.27%.

### 3.4. Comparison of ELISA and Ella Measurements of Galectin-3 Levels

**Figure 3:**
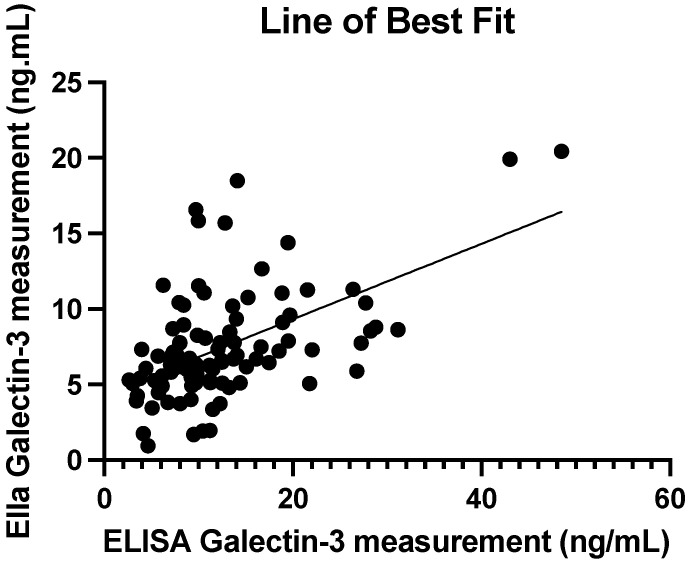
Best fit line of ELISA and Ella serum galectin-3 measurements with CV less than 10%. The Spearman’s correlation coefficient is r = 0.49 (*p* < 0.0001).

Spearman’s signed rank test was used to assess the strength of correlation between manual ELISA and automated Ella estimates of serum galectin-3 levels. The correlation coefficient was r = 0.49 (*p* < 0.0001), indicating a moderate correlation between ELISA and Ella estimates of serum galectin-3 levels. Figure 3 shows the line of best fit of the ELISA and Ella measurements. The spread of data around the best-fit line indicates a moderate positive correlation between ELISA and Ella measurements of serum galectin-3 levels.

**Figure 4:**
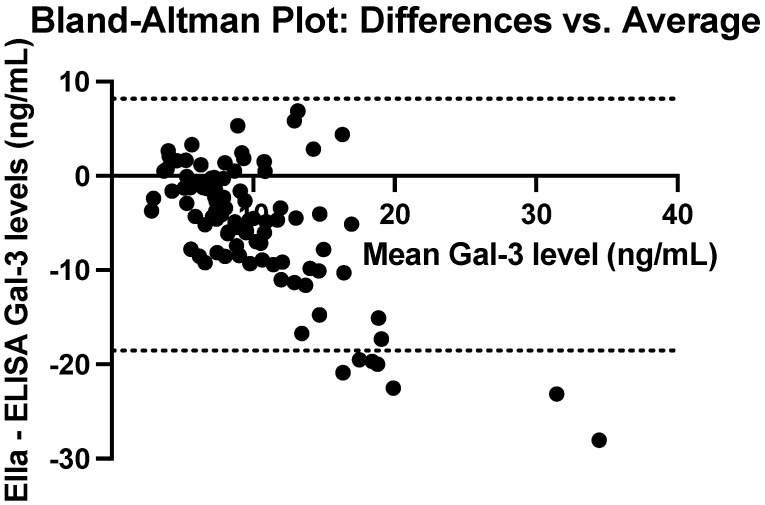
Bland-Altman Plot comparing the average serum galectin-3 levels between ELISA and Ella measurements, with the differences between the two techniques.

Serum galectin-3 levels measured using ELISA and Ella for the same patient sample were compared with a Wilcoxon signed-rank test. The mean difference was 5.19 ng/mL, with Ella resulting in significantly lower recorded serum galectin-3 levels than ELISA (*p* < 0.0001). The difference remained significant across all ELISA and Ella plates, with no significant difference found between plates. Even when only data points with a CV less than 5% (N = 56) are included in the sample, the Wilcoxon signed-rank test still yielded a significant difference between ELISA and Ella test results (*p* < 0.0001). Among samples with a CV less than 5%, there was a mean difference of 4.79 ng/mL. As the serum galectin-3 level increases, there seems to be a greater difference between the ELISA and Ella levels, as shown in the Bland-Altman Plot in Figure 4.

### 3.5. ELISA vs. Ella Measurements: Regression Line

**Figure 5:**
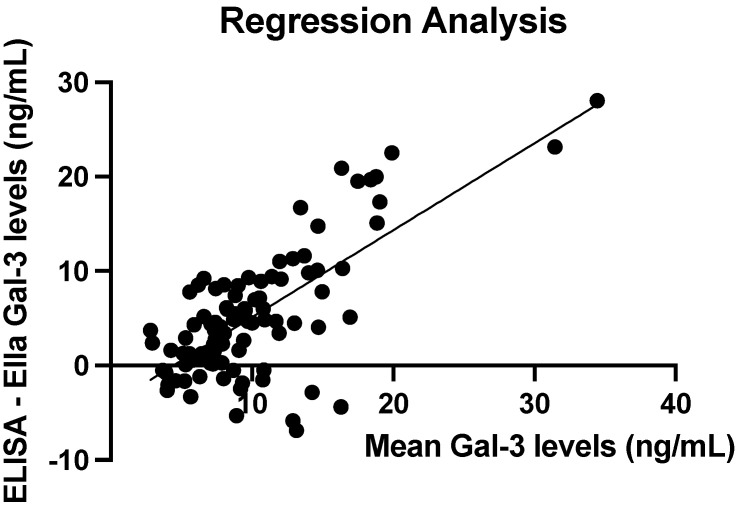
A fitted least-squares regression line between mean serum galectin-3 levels and the difference between ELISA and Ella values, with CV < 10%. Slope estimate = 0.92 ng/mL (*p* < 0.0001).

To quantify the apparent greater difference between ELISA and Ella measurements as serum galectin-3 levels increase, a least-squares regression line was fitted to the data. A significant relationship was found with, on average, an increase of 0.92 ng/mL (*p* < 0.0001) in the difference between ELISA and Ella measurements for each 1 ng/mL increase in mean serum galectin-3 levels. The $R^{2}$ value of this model is 0.5093, indicating that approximately 50.93% of the variance in the difference between ELISA and Ella measurements could be attributed to differences in the mean serum galectin-3 level. Figure 5 shows the fitted regression line with an increasing mean serum galectin-3 level associated with an increasing difference between the two techniques.

**Figure 6:**
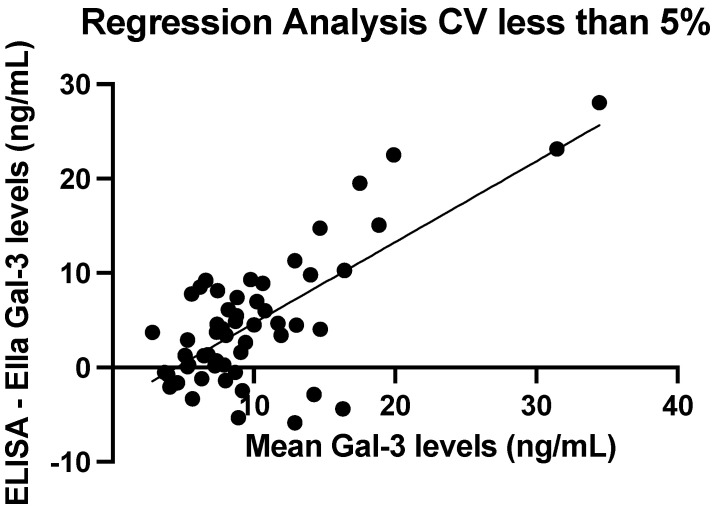
A fitted least-squares regression line between mean serum galectin-3 levels and the difference between ELISA and Ella values with CV < 5%. Slope estimate = 0.86 ng/mL (*p* < 0.0001).

When a least squares regression line is fitted to only measurements with a CV of less than 5%, a significant relationship is maintained. For each 1 ng/mL increase in the mean serum galectin-3 level between the two techniques, there is an approximately 0.86 ng/mL increase in the difference between ELISA and Ella values (*p* < 0.0001). The $R^{2}$ of this model is 0.5257, indicating that approximately 52.57% of the variance in the difference between ELISA and Ella measurements could be attributed to differences in the mean serum galectin-3 level. Figure 6 shows the fitted regression line with an increasing difference between the two techniques associated with an increasing mean serum galectin-3 level, in agreement with Figure 5.

### 3.6. Comparison of ELISA and Ella Measurements by Breast Cancer Stage

In 14. and N = 7, respectively, non-parametric analyses were still utilized to ensure a stringent analysis. Table 4 shows the results of those analyses with significant results highlighted in yellow. Specifically, the Spearman’s correlation between ELISA and Ella measurements indicates a moderately weak correlation in stages I and II, and a moderately strong correlation in stages III and IV. However, due to its small sample size, the correlation in the Stage IV subsample was not statistically significant. For the Wilcoxon Signed-rank test, there was a significant difference between ELISA and Ella measurements of serum galectin-3 levels in stages I, II, and III, with a mean difference of −5.34 ng/mL (*p* < 0.0001), −5.88 ng/mL (*p* < 0.0001), and −4.08 ng/mL (*p* = 0.0052), respectively. Additionally, there was a significant association between an increase in mean galectin-3 levels and an increase in the difference between ELISA and Ella levels, as indicated by the regression line slope for stages I and II (*p* < 0.0001). Furthermore, the $R^{2}$ of each regression model is reported in the last column of Table 4, showing the proportion of variability in differences between ELISA and Ella measurements that can be attributed to changes in the mean serum galectin-3 levels.

Additionally, to assess the potential impact of breast cancer stage on the differences between ELISA and Ella measurements, a multivariate regression was performed, and the association between breast cancer stage and differences in methodology measurements of serum galectin-3 levels was found to be statistically insignificant (*p* = 0.9441).

### 3.7. Comparison of ELISA and Ella Measurements by Breast Cancer Immunophenotype

This process was repeated within each immunophenotype subsample that possessed a large enough sample size for meaningful comparison. This included HER2 Enriched (N = 7), Luminal A (N = 17), Luminal B-like (N = 22), and Triple Negative (N = 20). Each immunophenotype was assessed for a normal distribution of ELISA and Ella values. Through utilizing a Shapiro-Wilk test, the distributions of both ELISA and Ella measurements in HER2-enriched and Luminal B-like were found to be non-normally distributed. The Shapiro-Wilk test did not find significant evidence of a non-normal distribution in the Luminal A and Triple Negative subsamples. However, due to the small sample sizes of these samples, non-parametric analyses were utilized to ensure a stringent analysis. Table 5 shows the results of those analyses. Specifically, Spearman’s correlation between ELISA and Ella measurements indicates a moderate correlation in HER2-enriched, Luminal A, and Luminal B-like subtypes, and a very weak correlation in the triple-negative subtype. However, the correlations in the HER2 Enriched and the Triple Negative immunophenotype were not statistically significant. There was a significant difference between ELISA and Ella measurement of serum galectin-3 levels in Luminal A, Luminal B-like, and Triple Negative subtypes, with a mean difference of −7.58 ng/mL (*p* = 0.0007), −4.42 ng/mL (*p* = 0.0014), and −5.49 ng/mL (*p* = 0.0056), respectively. Additionally, there was a significant association between an increase in mean galectin-3 levels and an increase in the difference between ELISA and Ella levels as indicated by the regression line slopes for HER2 Enriched (*p* = 0.0018), Luminal A (*p* < 0.0001), and Triple Negative (*p* = 0.0028) immunophenotypes. Furthermore, the $R^{2}$ value of each regression model is reported in the last column of Table 5, showing the proportion of variability in differences between ELISA and Ella measurements that can be attributed to changes in the mean serum galectin-3 levels.

Additionally, to assess the potential impact of breast cancer immunophenotypes on the differences between ELISA and Ella measurements, a multivariate regression was performed, and the association between immunophenotype and differences in methodology measurements of serum galectin-3 levels was found to be statistically insignificant (*p* = 0.9420).

### 3.8. Comparison of ELISA and Ella Measurements by Breast Cancer Histology

This process was repeated once again within each histology subsample that possessed a large enough sample size for meaningful comparison. This included Apocrine (N = 11), DCIS (N = 43), and Invasive Ductal (N = 18). Each histology sample was assessed for a normal distribution of ELISA and Ella values. A Shapiro-Wilk test found the ELISA and Ella measurements of DCIS to be non-normally distributed. There was no significant evidence found that the Apocrine and Invasive Ductal subsamples were not normally distributed. However, due to the small sizes of these samples, non-parametric analyses were utilized to ensure a stringent analysis. Table 6 shows the results of those analyses. Specifically, Spearman’s correlation between ELISA and Ella measurements indicated a statistically significant correlation for all three histologies, with a moderate correlation in the DCIS and Apocrine histologies, and a moderately strong correlation in the Invasive Ductal histology. There was a significant difference between ELISA and Ella measurement of serum galectin-3 levels in Apocrine, DCIS, and Invasive Ductal histologies, with a mean difference of −8.71 ng/mL (*p* = 0.0010), −4.38 ng/mL (*p* = 0.0003), and −2.41 ng/mL (*p* = 0.0066), respectively. Additionally, there was a significant association between an increase in mean galectin-3 levels and an increase in the difference between ELISA and Ella levels as indicated by the regression line slopes for DCIS (*p* < 0.0001) and Invasive Ductal (*p* = 0.0044) histologies. Furthermore, the $R^{2}$ value of each regression model is reported in the last column of Table 6, showing the proportion of variability in differences between ELISA and Ella measurements that can be attributed to changes in the mean serum galectin-3 levels within each histology group.

Additionally, to assess the potential impact of breast cancer histology on the differences between ELISA and Ella measurements, a multivariate regression was performed, and the association between histology and differences in methodology measurements of serum galectin-3 levels was found to be statistically insignificant (*p* = 0.2532).

## 4. Discussion

### 4.1. Study Findings

In concordance with previous studies, our findings confirm that the Ella instrument results in significantly lower CV values, making it a more reliable and precise measurement technique than traditional manual ELISA methods [48].

This study found that there is a significant difference between the manual ELISA and automated Ella results for serum galectin-3 levels in the same sample, with Ella, on average, resulting in significantly lower galectin-3 measurements than manual ELISA. This deviates from the results found in another study that compared different proteins in healthy controls using both Ella and manual ELISA. In that study, Ella accurately quantified protein levels consistent with manual ELISA [48].

A significant relationship was found between an increasing mean serum galectin-3 level and an increasing difference between ELISA and Ella levels. This is similar to previous findings on adiponectin levels using both ELISA and Ella, which found an increasing difference in ELISA and Ella measurements at higher adiponectin concentrations [50]. When the CV threshold is relaxed from less than 5% to less than 10%, the slope of the fitted least-squares regression increases. This suggests that additional, more variable data may show greater divergence between ELISA and Ella measurements at higher concentrations.

While our study is unable to make definitive conclusions as to the cause of the differences between the ELISA and Ella methods in measuring serum galectin-3 levels, there are several differences between the two techniques. For example, the microplate reader for manual ELISA utilizes absorbance data of the cells, while the Ella instrument uses fluorescence to calculate concentrations. This detection method makes Ella more sensitive to a wider range of protein concentrations, including lower concentrations than possible with manual ELISA. It is possible that this is a reason for the increasing differences in the two methods at higher galectin-3 concentrations. The standard curve for the Ella instrument is also preloaded and factory calibrated compared to the standard curve of ELISA, which must be created by the researcher and then utilized to calculate the concentrations of each sample. It is possible that this difference in standard curve generation impacts the concentration estimation at higher sample concentrations. The Ella instrument eliminated the need for multiple dilution series. However, if a different dilution series had been used to rerun the samples with higher galectin-3 levels in manual ELISA, there might have been higher concordance. Ultimately, this study assessed the concordance between the two methods, and we are unable to definitively attribute a specific cause to our findings.

### 4.2. Implications

Due to the rising clinical importance of galectin-3 in breast cancer pathways, automation of analysis methods is an important investment [33]. These findings underscore the need for standardization across galectin-3 assays, as researchers and clinicians may draw different conclusions solely based on the analytical method used. Additionally, if galectin-3 is used as a diagnostic or prognostic biomarker, different ELISA methods could result in different conclusions due to the divergence between methods. Because of the speed of analysis and higher precision of results, the automated Ella instrument is a promising tool for ensuring precise, replicable analysis of critical cancer biomarkers. The higher sensitivity, better reliability, and faster speed of the Ella instrument due to its automation have the potential to provide patients with quicker and more accurate results, even at low concentrations, in cases where there is good concordance between the Ella instrument and manual ELISA. This is the first study to date to compare the Ella instrument with traditional manual ELISA methods for quantifying serum galectin-3 levels in breast cancer patients. This marks a crucial step towards the possibility of utilizing automated methods to measure galectin-3 in breast cancer patients as a potential diagnostic, prognostic, and therapeutic target.

### 4.3. Limitations

Despite interesting differences between ELISA and Ella measurements of galectin-3 levels, it is not possible to discern from the results of this study whether Ella is underestimating ELISA or ELISA is overestimating Ella. Additionally, due to the lack of control samples, it is not possible to determine whether these findings are unique to breast cancer patients or indicative of a universal discrepancy between ELISA and Ella measurements of galectin-3 levels. Additionally, this sample size was constrained by sample availability in the Prisma Health Cancer Institute Biorepository. Repeating this analysis with a larger sample size would provide greater statistical power. If galectin-3 levels are to be used for diagnostic purposes or as therapeutic targets, more analysis must be done to determine which method provides the most accurate analysis.

### 4.4. Future Directions

Future studies should continue to analyze discrepancies in protein measurements to evaluate whether this is unique to the Ella instrument and galectin-3 levels or whether discrepancies are found among other instruments and proteins. Additionally, exploring whether these results are maintained for other cancer types is an important direction for future research. Due to the growing importance of galectin-3 in breast cancer research and the sample availability from the Prisma Health Cancer Institute Biorepository, this study focused on breast cancer patient samples. However, the presence of differences between the two methodologies necessitates further study as to whether these differences are maintained when assessing serum galectin-3 levels in healthy subjects. Previous studies have also shown differences in serum galectin-3 levels between varying immunophenotypes, histologies, and genetic mutation profiles [34,36,37,38]. Future studies should investigate whether novel automated ELISA techniques find similar differences in galectin-3 levels between differing breast cancer immunophenotypes and histologies. Furthermore, if prognostic or diagnostic tools are developed utilizing galectin-3 levels, analysis should be performed to ascertain whether the diagnostic and prognostic capacities are comparable between methods.

## 5. Conclusions

This is the first study comparing manual ELISA with the Ella instrument in measuring serum galectin-3 levels in breast cancer patients. The speed, reproducibility, and lower susceptibility to errors of the Ella instrument represent its promising potential as a tool for assessing biomarkers in breast cancer patients. However, understanding the cause of the differences between manual ELISA and Ella measurements is imperative for accurate assessment of galectin-3 levels in breast cancer patients.

## Figures and Tables

**Figure 1:**
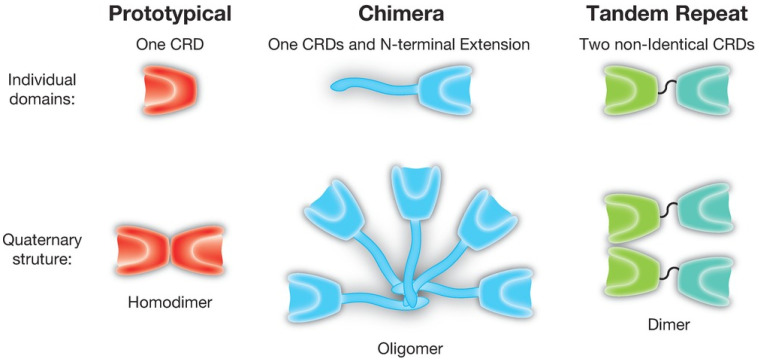
The three structural types of galectins, where CRD stands for Carbohydrate Recognition Domain. From Kamili, N.A. et al., 2016 [3].

**Figure 2:**
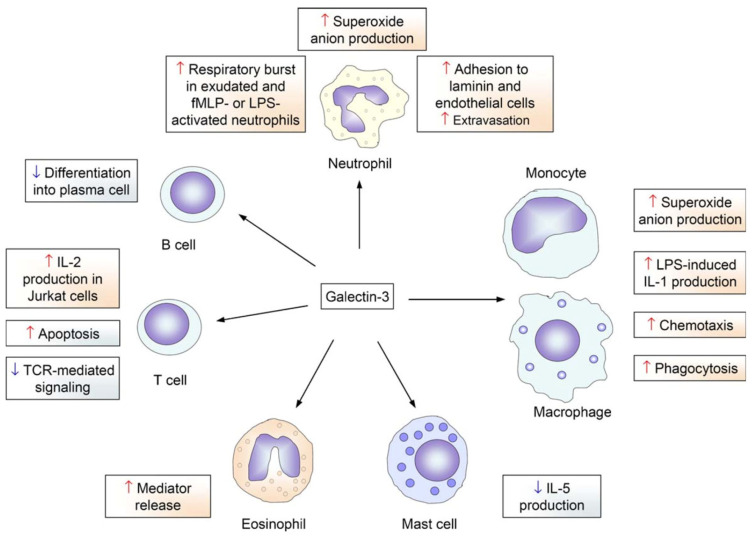
The role of galectins in immune system regulation. From Dumic, J. et al., 2006 [21].

**Table 1:** Clinical characterization of breast cancer patient samples included in this study by (a) stage, (b) immunophenotype, and (c) histology, where N represents the sample size.

(a). Stage	N = 115	(b). Immunophenotype	N = 79	(c). Histology	N = 115
I	47	HER2 Enriched.	7	Adenocarcinoma	6
II	42	Luminal A	20	Apocrine	15
III	17	Luminal B	5	Benign	4
IV	9	Luminal B-like	25	Carcinoma	5
		Triple Negative	22	Ductal Carcinoma in Situ	45
				Invasive Ductal	25
				Invasive Lobular	1
				Invasive Mammary	5
				Invasive Micropapillary	1
				Lobular	5
				Metaplastic	1
				Mucinous	2

**Table 2:** Summary statistics of galectin-3 concentrations measured using ELISA and Ella, where N represents the sample size.

	Mean (ng/mL)	SD (ng/mL)	Median (ng/mL)	IQR (ng/mL)	Min–Max (ng/mL)
ELISA (N = 95)	12.68	8.07	10.59	7.43–15.10	2.65–48.48
Ella (N = 95)	7.49	3.71	6.70	5.20–8.80	0.95–20.43
ELISA–Ella (N = 95)	−5.19	6.81	−4.32	−8.54–−0.30	−28.06–6.88

**Table 3:** Summary statistics of CV values with ELISA and Ella, where N represents the sample size.

	Mean (%)	SD (%)	Median (%)	IQR (%)	Min–Max (%)
ELISA CV (N = 115)	7.50	13.78	4.75	1.85–7.82	0.17–127.84
Ella CV (N = 115)	2.41	1.46	2.10	1.37–3.27	0.19–6.86

**Table 4:** Comparison of ELISA and Ella serum galectin-3 measurements by breast cancer stage, where N represents the sample size. Statistically significant results are highlighted in yellow.

Stage	Spearman’s Correlation Coefficient ^1^	Wilcoxon Signed Rank (ng/mL) ^2^	Regression Line Slope (ng/mL) ^3^	$R^{2}$ of the Regression Line ^4^
Stage I (N = 39)	0.39 (*p* = 0.0145)	−5.34 (*p* < 0.0001)	1.03 (*p* < 0.0001)	0.5992
Stage II (N = 35)	0.37 (*p* = 0.0270)	−5.88 (*p* < 0.0001)	0.97 (*p* < 0.0001)	0.5346
Stage III (N = 14)	0.78 (*p* = 0.0009)	−4.08 (*p* = 0.0052)	0.20 (*p* = 0.5152)	0.0361
Stage IV (N = 7)	0.71 (*p* = 0.0713)	−3.13 (*p* = 0.0781)	0.75 (*p* = 0.0892)	0.4698

^1^ Spearman’s Correlation Coefficient is a nonparametric test that measures the strength of the correlation between manual ELISA measurements of galectin-3 and Ella measurements of galectin-3. ^2^ The Wilcoxon Signed Rank test pairs each ELISA measurement of galectin-3 with the corresponding Ella measurement from the same serum sample to assess the mean difference between the two methods. ^3^ The Regression Line Slope estimates the average increase in the difference between the ELISA and Ella measurements of the same serum galectin-3 levels as the mean galectin-3 level increases. ^4^ The $R^{2}$ of the Regression Line estimates the proportion of variability in the difference between corresponding ELISA and Ella measurements of serum galectin-3 levels that can be attributed to a change in mean galectin-3 levels.

**Table 5:** Comparison of ELISA and Ella serum galectin-3 measurements by immunophenotype, where N represents the sample size. Statistically significant results are highlighted in yellow.

Immunophenotypes	Spearman’s Correlation Coefficient ^1^	Wilcoxon Signed Rank (ng/mL) ^2^	Regression Line Slope (ng/mL) ^3^	$R^{2}$ of the Regression Line ^4^
HER2 Enriched (N = 7)	0.54 (*p* = 0.2152)	−6.98 (*p* = 0.1094)	0.98 (*p* = 0.0018)	0.8805
Luminal A (N = 17)	0.59 (*p* = 0.0130)	−7.58 (*p* = 0.0007)	1.24 (*p* < 0.0001)	0.6696
Luminal B-like (N = 22)	0.43 (*p* = 0.0453)	−4.42 (*p* = 0.0014)	0.46 (*p* = 0.1769)	0.0892
Triple Negative (N = 20)	0.04 (*p* = 0.6970)	−5.49 (*p* = 0.0056)	1.25 (*p* = 0.0028)	0.4698

^1^ Spearman’s Correlation Coefficient is a nonparametric test that measures the strength of the correlation between manual ELISA measurements of galectin-3 and Ella measurements of galectin-3. ^2^ The Wilcoxon Signed Rank test pairs each ELISA measurement of galectin-3 with the corresponding Ella measurement from the same serum sample to assess the mean difference between the two methods. ^3^ The Regression Line Slope estimates the average increase in the difference between the ELISA and Ella measurements of the same serum galectin-3 levels as the mean galectin-3 level increases. ^4^ The $R^{2}$ of the Regression Line estimates the proportion of variability in the difference between corresponding ELISA and Ella measurements of serum galectin-3 levels that can be attributed to a change in mean galectin-3 levels.

**Table 6:** Comparison of ELISA and Ella serum galectin-3 measurements by histology, where N represents the sample size. Statistically significant results are highlighted in yellow.

Histology	Spearman’s Correlation Coefficient ^1^	Wilcoxon Signed Rank (ng/mL) ^2^	Regression Line Slope (ng/mL) ^3^	$R^{2}$ of the Regression Line ^4^
Apocrine (N = 11)	0.67 (*p* = 0.0233)	−8.71 (*p* = 0.0010)	0.59 (*p* = 0.1323)	0.2333
Ductal Carcinoma in Situ (N = 43)	0.43 (*p* = 0.0043)	−4.38 (*p* = 0.0003)	0.97 (*p* < 0.0001)	0.5301
Invasive Ductal (N = 18)	0.75 (*p* = 0.0004)	−2.41 (*p* = 0.0066)	0.73 (*p* = 0.0044)	0.4074

^1^ Spearman’s Correlation Coefficient is a nonparametric test that measures the strength of the correlation between manual ELISA measurements of galectin-3 and Ella measurements of galectin-3. ^2^ The Wilcoxon Signed Rank test pairs each ELISA measurement of galectin-3 with the corresponding Ella measurement from the same serum sample to assess the mean difference between the two methods. ^3^ The Regression Line Slope estimates the average increase in the difference between the ELISA and Ella measurements of the same serum galectin-3 levels as the mean galectin-3 level increases. ^4^ The $R^{2}$ of the Regression Line estimates the proportion of variability in the difference between corresponding ELISA and Ella measurements of serum galectin-3 levels that can be attributed to a change in mean galectin-3 levels.

## Data Availability

The data presented in this study are available in this article.

## Supplementary Materials

The following supporting information can be downloaded at: https://www.mdpi.com/article/10.3390/cancers17193206/s1, Table S1: ELISA and Ella Full Data.

## Abbreviations

The following abbreviations are used in this manuscript:

CCL2	Chemokine (C-C motif) Ligand 2
CD45	Leukocyte Common Antigen
CRD	Carbohydrate Recognition Domain
CV	Coefficient of Variation
DCIS	Ductal Carcinoma in Situ
ELISA	Enzyme Linked Immunosorbent Assay
ERK	Extracellular Signal-Regulated Kinase
Gal-3	Galectin-3
HER2	Human Epidermal Growth Factor Receptor 2
IL-6	Interleukin-6
IL-10	Interleukin-10
IQR	Interquartile Range
MCP-1	Monocyte Chemoattractant Protein-1
MEK	Mitogen-Activated Protein Kinase Kinase
NOTCH-1	Neurogenic Locus Notch Homolog Protein 1
PHCI	Prisma Health Cancer Institute
Raf	Rapidly Accelerated Fibrosarcoma
Ras	Rat Sarcoma
SD	Standard Deviation
TNF-α	Tumor Necrosis Factor Alpha
VEGF-A	Vascular Endothelial Growth Factor A
VEGFR2	Vascular Endothelial Growth Factor A Receptor 2
